# Circadian and Diurnal Regulation of Cerebral Blood Flow

**DOI:** 10.1161/CIRCRESAHA.123.323049

**Published:** 2024-03-15

**Authors:** Alastair J.S. Webb, Elizabeth B. Klerman, Emiri T. Mandeville

**Affiliations:** Department of Clinical Neurosciences, Wolfson Centre for Prevention of Stroke and Dementia, University of Oxford, United Kingdom (A.J.S.W.).; Department of Neurology, Massachusetts General Hospital, Boston (E.B.K.).; Division of Sleep and Circadian Disorders, Department of Medicine, Brigham and Women’s Hospital, Boston, MA (E.B.K.).; Division of Sleep Medicine, Harvard Medical School, Boston, MA (E.B.K.).; Departments of Radiology and Neurology, Neuroprotection Research Laboratories, Massachusetts General Hospital, Harvard Medical School, Boston (E.T.M.).

**Keywords:** blood pressure, cardiovascular diseases, cerebrovascular circulation, heart disease risk factors, risk factors

## Abstract

Circadian and diurnal variation in cerebral blood flow directly contributes to the diurnal variation in the risk of stroke, either through factors that trigger stroke or due to impaired compensatory mechanisms. Cerebral blood flow results from the integration of systemic hemodynamics, including heart rate, cardiac output, and blood pressure, with cerebrovascular regulatory mechanisms, including cerebrovascular reactivity, autoregulation, and neurovascular coupling. We review the evidence for the circadian and diurnal variation in each of these mechanisms and their integration, from the detailed evidence for mechanisms underlying the nocturnal nadir and morning surge in blood pressure to identifying limited available evidence for circadian and diurnal variation in cerebrovascular compensatory mechanisms. We, thus, identify key systemic hemodynamic factors related to the diurnal variation in the risk of stroke but particularly identify the need for further research focused on cerebrovascular regulatory mechanisms.

Cardiovascular events occur more frequently in the morning between 6 am and 12 noon,^1^ are more common in patients with a mismatch between endogenous circadian rhythm and the environment,^2^ are increased in patients with abnormal diurnal blood pressure changes,^3^ and recovery from stroke may be worse if a stroke occurs at night.^4^ This reflects behavior, environmental rhythms, and endogenous circadian variation in physiological mechanisms from blood clotting^5^ to cardiac arrhythmias.^6^ However, although there has been extensive study of diurnal variation (ie, the nonlinear combination of endogenous circadian and evoked changes from sleep/wake state, activity levels, posture, eating, and other factors) in systemic hemodynamics,^7,8^ there has been limited focus on diurnal variation in cerebral blood flow (CBF) and its dependence on both systemic physiology and cerebrovascular regulatory mechanisms.

Endogenous circadian variation increases survival probability by adapting physiological needs to anticipate the influence of the earth’s 24-hour cycle, with diurnal variation in systemic blood pressure as the archetypal cardiovascular example. The associated cardiovascular risk may, therefore, result either from normal hemodynamic diurnal variation, exacerbated in later life by both the increased absolute cardiovascular risk and impaired compensatory mechanisms; from pathologically abnormal circadian rhythms; and from asynchrony between endogenous circadian rhythms and the environment as we become less dependent on the natural environment. Outcomes associated with diurnal rhythms may potentially be altered by changing or minimizing some of the factors (eg, timing of activity, posture changes, or eating) that determined both these diurnal cycles and how they interact with the underlying endogenous circadian rhythms.

## CEREBROVASCULAR RISKS ASSOCIATED WITH CIRCADIAN AND DIURNAL RHYTHMS

Although early studies identified an evening peak in cerebrovascular events,^9^ the majority of studies have demonstrated a strong morning peak between 6 am and 12 noon,^10–12^ with an overall increased risk of ischemic stroke of 49% compared with other times of day^1^ and an even stronger association with hemorrhagic stroke.^1^ The high incidence of cerebrovascular events is specifically associated with a surge in blood pressure soon after waking,^13^ with a time-of-day bimodal peak in incidence in some cohorts^4,14^ consistent with the bimodal variation in blood pressure. However, studies of diurnal variation in the etiological subtype of ischemic stroke have been less consistent. There is a consistent increase in morning cardioembolic and large artery strokes but a greater than expected incidence of patients waking up with lacunar strokes,^15^ potentially due to an increased nocturnal risk when blood pressure is low.^15–17^ The correlation between blood pressure and risk implies that diurnal variation in CBF and its regulation is critical to both the triggering of stroke and the brain’s resilience to variation in blood flow. This correlation, thus, provides a pathological marker of diurnal hemodynamic rhythms and a marker of the pathophysiological distinction between cardioembolic stroke,^18^ large vessel disease,^19^ and small vessel occlusion.^20^ However, 50% of strokes of unknown, mixed, or rarer etiology (dissection, prothrombotic states, vasculitis, etc) may have differing diurnal rhythms.^21^

In addition to stroke incidence, cross-sectional analysis of large acute stroke imaging databases has identified a reduced ratio of stroke penumbra to core in strokes that occur in the night,^22^ with a worse outcome after endovascular treatment^23^ and an increased risk of early neurological deterioration,^24^ indicative of an interaction with resilience to vessel occlusion, while the probability of death or a poor outcome is greater for strokes occurring during the night.^4^

Although acute stroke is the clearest manifestation of cerebrovascular disease, resulting in both physical disability and cognitive impairment,^25^ chronic cerebrovascular disease may also be exacerbated by circadian and diurnal influences on CBF. Cerebral small vessel disease, manifested as chronic white matter hyperintensities, microbleeds, dilated perivascular spaces, and lacunar infarcts,^26^ is associated with an increased risk of acute events,^27^ causing 30% of ischemic stroke, 80% of hemorrhagic stroke, and a 40% risk of all-cause dementia, late-onset refractory depression,^28^ and a markedly increased risk of functional dependence, late-life multimorbidity, and frailty.^29^ This is particularly associated with hypertension and strongly influenced by differences in diurnal rhythms between individuals with increased severity of disease in patients with excessive nocturnal blood pressure.^30^

## MECHANISMS OF CIRCADIAN AND DIURNAL CONTROL OF PHYSIOLOGY

Diurnal variation in CBF results from the integration of cerebrovascular mechanisms with circadian variation in systemic blood pressure due to the external environment, our behavioral responses, and endogenous circadian rhythms. The cellular circadian clock and its synchronization with the external world via environmental zeitgebers such as light have been dissected in great detail and extensively reviewed elsewhere.^31^ Briefly, the central cellular clock is driven by heterodimerization of the circadian locomotor output cycles kaput (CLOCK) and BMAL1 (basic helix-loop-helix ARNT-like protein 1) proteins that promote gene transcription affecting up to 10% of cellular proteins^32^ through ebox targets. This includes transcription of 5 core clock genes that provide negative transcriptional/translational feedback loops, with binding of 3 PERIOD (period circadian regulator) proteins (PER1-3) and 2 cryptochromes (CRY1/2), together with casein kinase (CK) to form a complex that is phosphorylated, resulting in translation to the nucleus. Similar negative feedback loops work in parallel through proteins such as Rev-erb (nuclear receptor subfamily 1 group D). Together, these prevent CLOCK/BMAL1-dependent transcription and heterodimerization and BMAL1 transcription.^31^ This reverberating cellular cycle produces a spontaneous rhythm of gene transcription with a period of slightly longer than 24 hours in humans and other day-active mammals and slightly shorter in rodents.

These cellular circadian rhythms do not have the same cycle length within^33^ and across tissues and may lose phase synchrony,^34^ requiring synchronization to each other and to the external environment. The principal zeitgeber is light, detected by photosensitive retinal ganglion cells,^35,36^ and transmitted via the retinohypothalamic tract^37^ to the suprachiasmatic nucleus (SCN) in the hypothalamus, the master clock in mammals.^38^ The SCN then synchronizes intrinsic cellular circadian rhythms throughout the brain and the body, first locally via both chemical signals^39^ and neuronal connections,^40^ and then throughout the body via mechanisms that are not fully resolved but include hormonal release from the pituitary gland,^41^ the pineal gland, and modulation of the descending autonomic nervous system.

### Integrated Control of Systemic Hemodynamics and Cerebral Perfusion

Maintenance of cerebral perfusion is one of the central purposes of the cardiovascular system, receiving up to 20% of cardiac output despite weighing only 2% of body weight.^42^ In common with the kidneys, the brain has a low-resistance vascular bed optimized to receive high blood flow continuously throughout the cardiac cycle to meet the constant demand of energy-hungry, high-metabolic activity tissue, resulting in a close following response of CBF on systemic blood pressure. However, the cerebral circulation is not a passive recipient but actively modifies vascular resistance to integrate this perfusing pressure with the needs of the brain,^43^ predominantly due to a distal change in tone^44^ both to adapt CBF to changes in blood pressure (autoregulation),^45^ to regional neuronal demand (neurovascular coupling),^46^ and to global stimuli such as the Pco_2_, pH, and oxygen (cerebrovascular reactivity [CVR]).^47^ Nonetheless, the low resistance of the cerebral circulation and its close proximity to the heart means that the brain is still particularly exposed to variations in blood pressure, a cost of the need to maintain continuous perfusion while minimizing pulsatility in the microcirculation.^48^

CBF is, therefore, strongly affected by both cardiac output and total peripheral resistance, the 2 key determinants of systemic perfusing pressure. An acute fall in cardiac output directly results in a fall in CBF,^49,50^ even in the context of limited changes in arterial blood pressure, which results in a greater increase in peripheral vascular resistance than cerebrovascular resistance^51^ to maintain blood pressure and cerebral perfusion. A chronic reduction in cardiac output, such as due to heart failure, is also associated with chronically decreased CBF and improved by treatment with ACE inhibitors^52^ or heart transplantation.^53^ Similarly, an increase in peripheral vascular resistance, particularly due to neurohumoral stimuli such as adrenaline or sympathetic innervation as occurs in the early morning, increases mean blood pressure and, thus, cerebral perfusing pressure although this is partially counteracted by concomitant increases in cerebrovascular resistance.^50^ In addition to increases in the mean blood pressure, the pulsatile arterial waveform is also dampened at points of impedance (eg, the carotid bifurcation) and regions of vessel tortuosity such as the carotid siphon in humans^48^ and the rete mirabile in other mammals,^54^ with further dampening of the waveform as the total area of the vascular system increases and resistance decreases with the transition to arterioles and capillaries. Finally, the pulsatility of the arterial waveform also depends on the length of the cardiac cycle, with increasing pulsatility at lower heart rates exacerbating transmission through stiff vessels to the brain^55^ and potentially increasing the risk of chronic cerebrovascular disease in older patients.^56,57^

Autoregulation aims to maintain stable CBF across a broad range of blood pressures although the classical formulation of cerebral autoregulation as constant blood flow across a wide range of blood pressure was defined in anesthetized animals^58–60^ and may not accurately reflect the awake state in humans although it is a readily comprehensible formulation to understand cerebrovascular injury during hypotension^61^ or hypertension.^62^ However, most blood pressure fluctuations are rapid or rhythmic,^63^ and the autoregulatory response is not instantaneous.^64^ Therefore, a modern formulation of cerebral autoregulation is defined by the speed and efficiency by which CBF returns to normal in the face of a change in blood pressure.^65–68^ The mechanisms of autoregulation have been reviewed in detail^43^; there are 3 key mechanisms: a myogenic mechanism, whereby smooth muscle vascular tone rapidly changes in direct response to intravascular pressure; metabolic mechanisms controlled by chemical influences mediated by the endothelium (such as nitric oxide [NO]) or acting on vascular smooth muscle cells (VSMCs; such as CO_2_-dependent pH changes), which adjusts both basal vasomotor tone and the gain of the vasomotor response to other stimuli;^69^ and autonomic innervation of the vessels. The latter 2 processes show both intrinsic diurnal variation and are dependent on endothelial function, particularly NO release from the endothelium which then induces VSMC relaxation.^70^

Cerebral perfusing pressure is also modified by CVR, the vasomotor response to global stimuli such as Pco_2_. Cerebral vasodilatation in response to increased carbon dioxide is likely mediated by a decrease in extravascular pH and its influence on VSMCs^71^ and measurable by the blood flow response to inhaled carbon dioxide or respiratory maneuvers with transcranial ultrasound or magnetic resonance imaging,^72^ with similar but smaller responses to a low Po_2_ or tissue acidosis. This mechanism interacts with multiple vasodilatory and vasoconstrictor stimuli, including the autoregulatory response to blood pressure changes, with reduced autoregulatory gain as carbon dioxide increases.^73^ These vasodilatory tests, thus, provide an index of both VSMC function and underlying activity of the endogenous vasodilatory mechanisms, depending on the endothelial release of NO and subsequent relaxation of VSMCs, as demonstrated by their modification by drugs that donate NO,^74^ increase intracellular cGMP,^72^ or increase the response to carbon dioxide.^75^

Finally, local neuronal demand induces a potent vasodilatory stimulus to increase supply to meet the extra metabolic demand. Although the exact mechanisms of neurovascular coupling remain disputed, they are likely mediated by rapidly acting vasoactive molecules (such as NO) from neurons and astrocyte endfeet, hyperpolarization of local endothelial cells in nearby capillaries, and rapid backpropagation of signals up the capillary tree to tonically activate pericytes and VSMCs in local arterioles and first- and second-order capillaries.^76^ Circadian and diurnal influences on neurovascular coupling may, therefore, interact with circadian and diurnal effects on CVR and autoregulation, which depends on the same vasodilator effectors^77^ and partially on endothelial function.

### Circadian and Diurnal Rhythms in Systemic Determinants of CBF

#### Blood Pressure: The Archetypal Diurnal Rhythm

Systemic blood pressure is perhaps the most well-investigated physiological diurnal rhythm in humans^78^ and one of the most studied diurnal risk factors for disease.^79^ Blood pressure is normally higher during the day to meet the demands of an active, mobile organism and declines at night by 10% to 20% (dipping). In addition to the strong correlation between the diurnal peak in cerebrovascular risk with the diurnal peak in blood pressure,^1^ both a lack of or a reversal of this normal dipping behavior^30^ and extreme dipping below 20% of daytime levels^3^ are associated with an increased risk of all cardiovascular events.^80^ Indeed, nocturnal blood pressure is the strongest hemodynamic predictor of future cerebrovascular events,^81^ exceeding the predictive value of daytime blood pressure; it is, therefore, an additional treatment target in both European^82^ and international blood pressure guidelines.^83^ In contrast, the association between extreme dipping profiles and cardiovascular risk is not consistent but may interact with age with a relationship only in older patients.^3^ In this group, there is aortic stiffening, reduced vessel elasticity, and diastolic pressure fall. Given the greater resulting transmission of pulsatile pressure to the brain, this may explain some of the greater risk of lacunar strokes occurring on wake-up compared with other stroke etiologies, reflecting hypoperfusion events in those patients with low nocturnal pressures and stiff vessels (Table).^84^

**Table.**
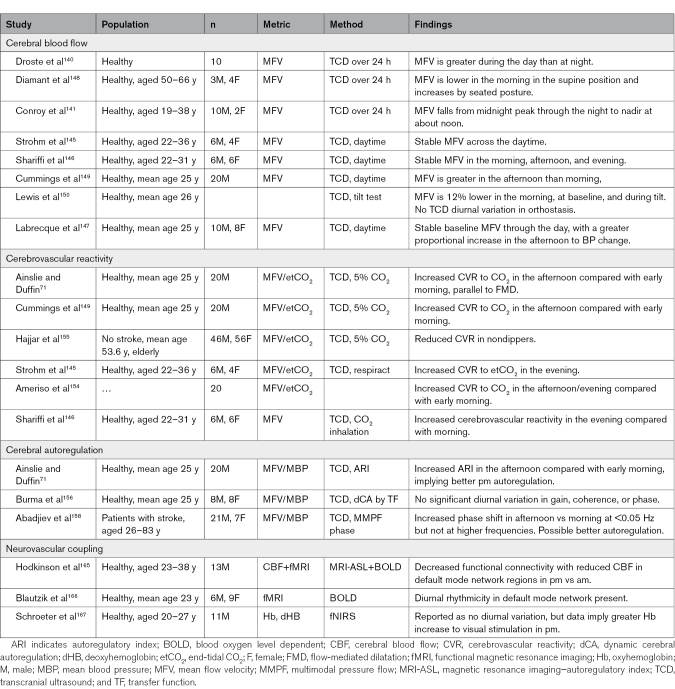
Physiological Studies in Humans Assessing Diurnal Variation in Control of Cerebral Blood Flow

The normal nocturnal fall in blood pressure is particularly associated with a fall in heart rate resulting in a fall in cardiac output.^85^ This is dependent on both the endogenous cardiac myocyte circadian clock,^86^ which has a potent influence on heart rate, combined with circadian variation in the autonomic nervous system, with a strong parasympathetic vagal drive at night, under the influence of the SCN.^87,88^ As awakening approaches, there is a surge in sympathetic drive, associated with a release of pituitary-dependent sympathomimetic hormones, including cortisol.^89^ This is followed by arousal and awakening and the transition to the upright posture. These trigger the morning surge in blood pressure, the magnitude of which is reflective of sympathetic activity, is associated with an increased risk of cardiovascular events,^90^ and is associated with a lower CBF in older adults.^91^ Subsequently, blood pressure falls in the mid-morning, particularly in patients on antihypertensive medication, often with a bimodal pattern during the day driven by a postprandial early afternoon fall in blood pressure.^92^ This diurnal variation in blood pressure determines the input pressure to the brain, providing greater flow in active periods, but with an increased risk of hypoperfusion during the night.

Key evidence for the role of the autonomic innervation of the heart in normal dipping, under the influence of both the SCN and peripheral autonomic reflexes, arises from patients with high cervical spinal cord injuries versus thoracic spinal cord injuries,^93^ diurnal variation in microneurography of sympathetic nerves,^88^ and loss of dipping in patients with dysautonomia.^94^ First, sympathomimetic neurohumoral modulation of diurnal blood variation is seen with morning adrenergic surges^89,95^ and normalization of a nondipping profile with nighttime administration of melatonin.^96^ Second, in circadian misalignment in a model of jet lag, the heart rate determinant of cardiac output shows a mismatch between the PR interval (indicative of the endogenous cardiac myocyte clock) and the heart rate (as driven by autonomic drive).^6^ Third, the nocturnal dip in blood pressure itself varies by the timing of sleep^97,98^ and by periodic nighttime arousals. In pathological circumstances, and in rapid eye movement sleep (ie, one of the stages of sleep) when blood pressure rises, there is increased sympathetic nerve activity and prohypertensive hormone release.^99^ Consistent with this parasympathetic/sympathetic imbalance, renal sympathetic denervation has a specific effect in reducing nocturnal BP. Heart rate changes, however, are also due to reflex changes to changes in venous return. The normal decrease in the body’s oxygen consumption at night is expected to reduce venous return from skeletal muscle and the gastrointestinal tract and reduce afferent activity of atrial stretch receptors that sense cardiac filling, thereby reducing the contribution of this reflex to promote tachycardia and permitting a predominance of parasympathetic influence on heart rate and any endogenous effects of a cardiac myocyte circadian clock.

However, chronotherapy that aims to modify blood pressure by drug dosing at different times of day has produced mixed results. The Hygia Chronotherapy Trial^100^ was associated with a marked reduction in cardiovascular events, but this has not been confirmed. This was a long-term outcome trial within a single regional service but lacked many of the features expected of a modern clinical trial (eg, independent oversight, transparent randomization procedures) and was associated with an unrealistic clinical benefit, including a marked reduction in noncardiovascular mortality. In contrast, the rigorously performed TIME (Treatment in Morning versus Evening) trial^101^ found no difference in clinical outcomes with morning versus nighttime dosing. This is consistent with limited effects on measured ambulatory blood pressure at different times of day in the HARMONY (Hellenic-Anglo Research into Morning or Night Antihypertensive Drug Delivery) trial.^102^

#### Circadian and Diurnal Cardiac Rhythms

The intrinsic circadian rhythm of myocytes has been extensively studied, with both cell models and ex vivo whole heart physiological models. Ex vivo myocyte cultures demonstrate circadian rhythms in the core clock genes (CLOCK, BMAL1, PER1, etc), driving circadian oscillation in 10% of the cardiac myocyte transcriptome.^32^ This circadian variation switches the cell between a predominantly catabolic state of high ATPase activity, protein synthesis, and fatty acid oxidation in periods of activity to periods of fatty acid storage and tissue repair and regeneration at times of rest. This includes rhythmic transcription of e4bp4,^103^ key ion channels responsible for the key transmembrane currents, particularly Kv1.5 and Kv4.2 potassium channels,^104^ and the sodium HCN4 channel responsible for the funny current that is key to determining the endogenous sinoatrial nodal rate.^105,106^ Diurnal oscillations are evident in the classical components of the ECG at night with lengthening of the PR interval, as wells as the RR interval and the QT interval. The cardiomyocyte clock is not directly driven by light in humans, but evidence for an autonomic mechanism for synchronization with the SCN is demonstrated by ex vivo resetting of the clock by noradrenaline^107^ or angiotensin II,^108^ addition to culture media.

**Figure 1.**
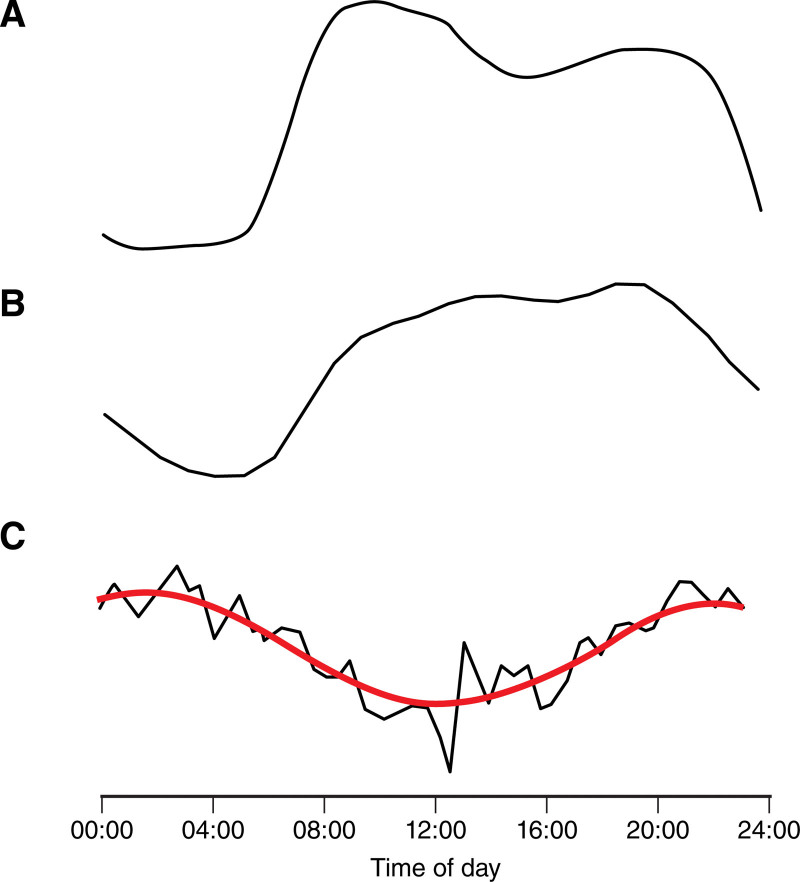
**Haemodynamic diurnal variation.** Diurnal variation at rest in (**A**) mean arterial pressure (blood pressure), (**B**) heart rate, and (**C**) middle cerebral artery velocity. (**C**) is adapted from Conroy et al^139^ with permission. Figure credit: Ben Smith.

Clinically, the nocturnal resting heart falls in humans to a similar degree to the decline in blood pressure (by ≈15%–20%; Figure 1).^109^ This is limited by autonomic denervation, evident in transplanted hearts, cardiomyocyte monolayers,^107^ or explanted hearts^110^ but is not abolished.^111^ Similarly, SCN ablation and pharmacological autonomic system blockade also reduce diurnal variation in heart rate,^112^ correlated with the diurnal variation in sympathomimetic hormone levels^89^ and sympathetic nerve activity.^87,88^ Asynchrony between the endogenous cardiomyocyte rhythm and the autonomic nervous system is demonstrated by a model of human jet lag in which a rapid phase shift in light results in rapid synchronization of heart rate under the influence of the synchronized SCN and (diurnal) behaviors that affect heart rate but a more gradual resynchronization of the PR interval, reflective of the intrinsic cardiomyocyte clock.^6^ This variation in heart rate may result in a differential effect on systolic and diastolic flow as the heart rate affects cerebral pulsatility,^55^ with a reduced diastolic perfusing pressure at the end of the cardiac cycle. This may also have effects on glymphatic drainage of the brain, which is driven by arterial pulsatility, which demonstrates an important diurnal variation and relationship with sleep,^113^ dealt with in another article within this compendium. We could identify no published studies of diurnal variation in cerebral pulsatility at this time. Abnormal dipping of heart rate is also associated with increased cardiovascular risk, comparable to abnormal dipping behavior of blood pressure.^114^

There is less direct evidence for circadian or diurnal variation in contractility and stroke volume, beyond the circadian oscillation in metabolic activity^86^ and transcription of contractile proteins,^110^ although this may be partially inferred from neurohumoral levels that have a morning peak in concentration and have a direct positive inotropic effect on the heart.

Circadian and diurnal cardiac rhythms may also predispose to arrhythmia. Sinus bradycardia and sinus pauses are particularly common at night due to the vagal drive. This can predispose to nocturnal heart block^115^ and to an increased probability of atrial fibrillation (AF),^116^ with a greater probability of reversion to sinus rhythm in the morning. This phenomenon could be partially responsible for larger relative ischemic cores in the early hours,^22^ as AF-related occlusion may affect larger vessels and multiple vascular territories (when AF-related, more severe large vessel occlusion could predominate). However, a greater burden of AF at night may also exacerbate intermittent hypoperfusion of the brain synergistically with lower mean blood pressure, as suggestive by greater imaging and cognitive decline in AF, independent of embolic events.^108^

#### Blood Viscosity

Blood viscosity, a key determinant of CBF and blood flow velocity,^117^ also displays diurnal variation. More than 5 decades ago, Seaman et al^118^ reported that hematocrit, total protein, hexosamine, protein-bound carbohydrate, and blood viscosity were high early in the morning. During diurnal cycles of human inactivity and bed rest, blood plasma volume is reduced by dehydration as gravitational forces shift plasma constituents to interstitial spaces and increase water excretion.^119^

Elevated blood viscosity increases blood flow resistance, induces platelet aggregation, and presents a risk factor for cerebrovascular stroke. Blood viscosity minimally impacted CBF in healthy young subjects whose autoregulation was not disrupted,^120^ consistent with a preclinical result in healthy animals showing a nonsignificant trend for decreased CBF at elevated viscosity. However, under high CBF states such as hypercapnia and chronic hypoxia, blood viscosity affects CBF,^121^ presumably due to an attenuated capacity for compensatory vasodilation. Therefore, blood viscosity may contribute to the diurnal variation in stroke risk in patients with comorbid states and disrupted autoregulation.

Several studies have also demonstrated that blood viscosity, especially in the diastolic cardiac phase, is associated with small vessel strokes such as lacunar stroke and silent ischemic lesions more than other stroke subtypes.^122,123^ Moreover, blood viscosity was correlated with early neurological deterioration in patients with lacunar infarct.^124^ The association of small vessel stroke with viscosity might result from the dependence of blood viscosity on vessel size—smaller vessels have a lower shear rate and, therefore, higher viscosity than large arteries and a greater tendency to aggregate red blood cells,^124^ thus contributing to the potential association between an increased risk of lacunar infarction at night in patients with reduced perfusing pressures.^15^

#### Vasomotor Tone

Flow-mediated dilatation studies of the systemic circulation in humans demonstrate increased basal vasomotor tone in the morning with impaired flow-mediated dilatation, concurrent with the morning surge in blood pressure and the increased incidence of stroke,^125^ predominantly reflecting alpha-adrenergic sympathetic nerve activity,^88,126^ with parallel effects causing impaired dilatation of cutaneous skin responses.^95^ In animal studies, there is ex vivo circadian variation in aortic tone, indicative of an endogenous circadian rhythm in rats with dopamine beta-hydroxylase deficiency in whom this is not dependent on adrenergic stimulation.^127^ This is consistent with circadian variation in both core clock genes^128^ and their transcriptional targets in mouse aorta,^129^ with integration between intrinsic circadian rhythms in vascular tissue and synchronization via the central clock.

The vasomotor tone is dependent on the release of vasodilatory molecules from the endothelium, inducing relaxation of VSMCs,^130^ and vasoconstrictors released by either the endothelium (endothelin-1 or angiotensin 2),^131^ circulating in the blood (adrenaline) or released by autonomic nerves innervating the vessels. These act on the VSMCs to decrease or increase vasomotor tone in the vessels and control peripheral vascular resistance. Mutation of the clock gene Per2 results in impaired systemic endothelial function with a reduced aortic endothelial response to acetylcholine in mice, particularly at the transition between the inactive sleep phase and the active phase, and reduced release of NO,^132,133^ while loss of Bmal1 results in loss of circadian rhythms in response to induced vasoconstriction, likely via ROCK2 (Rho associated coiled-coil containing protein kinase 2),^134^ as well as independent circadian variation in NO synthase activity.^130^ Reduced NO is associated with reduced circadian variation in vascular function,^135^ while obesity suppresses both NO activity, circadian gene expression, and endothelial function.^136^ Furthermore, there is circadian variation in vasoconstrictor hormones, such as endothelin-1.^137^ Overall, although there are endogenous clocks in both endothelial cells and VSMCs, circadian variation in systemic vasoconstrictor hormones and autonomic innervation is the predominant determinant of circadian variation in vasomotor tone.

### Circadian and Diurnal Rhythms in Cerebrovascular Function

#### Cerebral Blood Flow

CBF in the rat is increased in the active nocturnal phase of the cycle^138,139^ and significantly lower in the rest phase in awake animals although its dependence on activity versus endogenous rhythms is less clear. In humans, limited studies demonstrate a similar pattern of reduced blood flow and blood flow velocity during the night (Figure 1),^140,141^ with a ≈20% reduction at its nadir from its peak, similar to changes in blood pressure. CBF is more reduced in patients with obstructive sleep apnea^142^ and rises during rapid eye movement sleep,^143,144^ indicative of the importance of diurnal arousal versus endogenous circadian rhythms. However, studies are inconsistent, with some studies demonstrating a relatively stable middle cerebral artery velocity throughout the day,^145–147^ except when stimulated by activity,^148^ but the majority demonstrating a lower middle cerebral artery velocity in the morning compared with the evening at rest.^141,149,150^ This is in contrast to systemic perfusing pressure. Instead, the increase in CBF velocity appears to reach its peak at approximately midday, with an elevated evening CBF velocity compared with the morning and a ≈6-hour phase delay with the diurnal oscillation of blood pressure and core body temperature (Figure 1).^141^ This persists even in people who remain awake throughout the 24-hour cycle.^141^ However, these results are strongly dependent on activity, with an increased middle cerebral artery velocity induced by activity, dominating this underlying rhythm, although the increased propensity for morning syncope,^151^ reduced orthostatic tolerance in the morning,^64,150^ and the diurnal variation in stroke incidence^4^ indicate a role for underlying physiological mechanisms in adapting to diurnal behaviors.

#### Cerebrovascular Function

**Figure 2.**
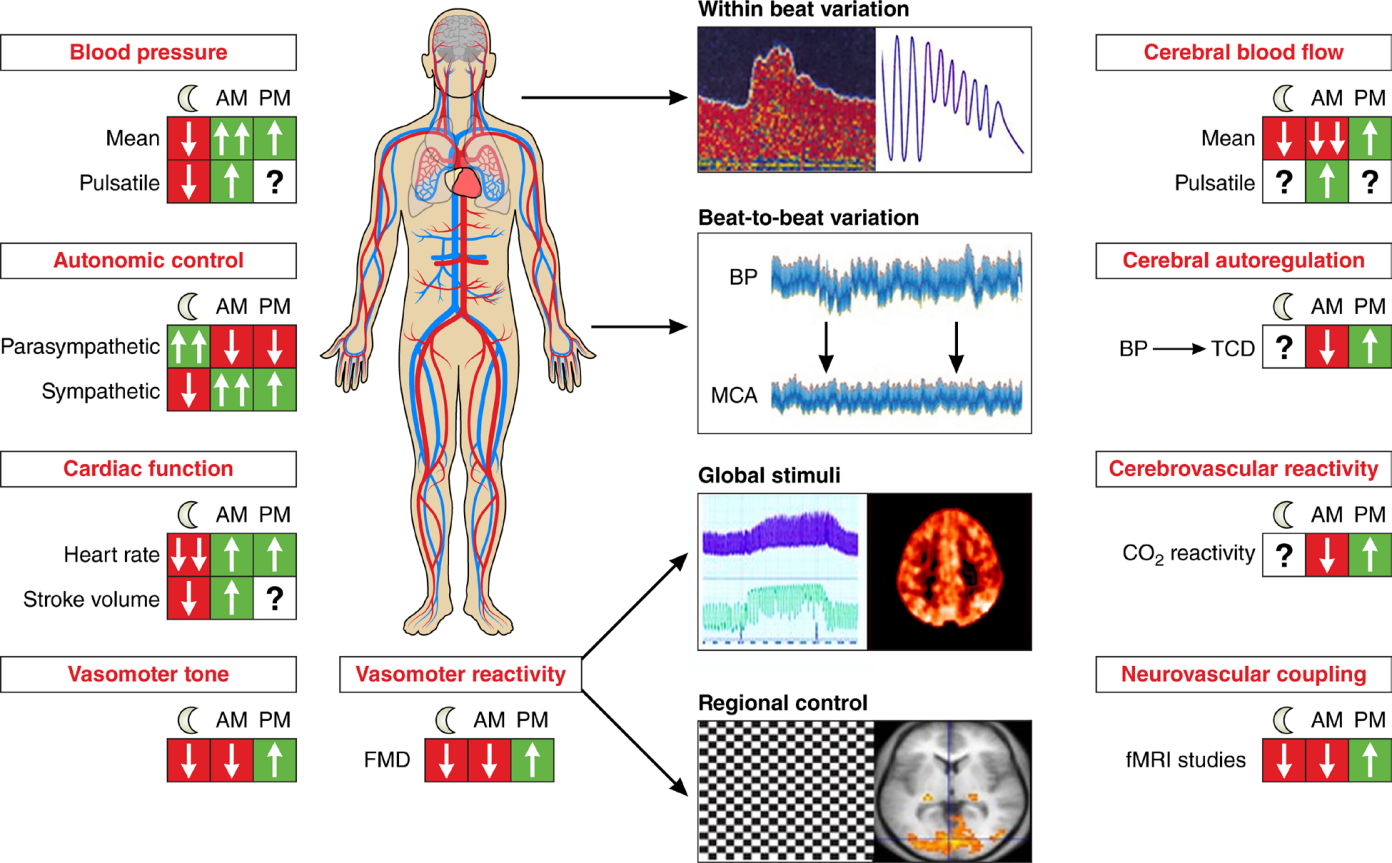
**Diurnal variation in systemic and cerebral mechanisms determining cerebral perfusion.** The direction of change in each index is shown for night, morning, and afternoon, with the magnitude of change indicated by the number of arrows. Figure credit: Ben Smith. BP indicates blood pressure; FMD, flow-mediated dilatation; fMRI, functional magnetic resonance imaging; MCA, middle cerebral artery; and TCD, transcranial ultrasound.

In contrast to the relatively extensive evidence for circadian variation in systemic vasomotor tone, there are limited data on circadian variation in cerebrovascular tone. In animals, there is rhythmicity in myogenic tone in the context of experimental subarachnoid hemorrhage,^152^ but measures of nocturnal cerebrovascular endothelial function in humans are challenging. The most direct index is reactivity to inhaled carbon dioxide during measurement of CBF by transcranial ultrasound or blood flow sensitive brain imaging (blood oxygen level dependent or arterial spin labeling–magnetic resonance imaging, perfusion CT, etc), but this is impractical in sleeping subjects. Furthermore, despite functional similarities between cerebral and systemic vascular endothelium during the day, this cannot be assumed at night. In a cohort of healthy volunteers, both CVR and flow-mediated dilatation in the forearm were more impaired in the morning than the afternoon,^153^ confirmed by studies demonstrating lower CVR in the morning to carbon dioxide compared with the afternoon,^149,153,154^ and reduced cerebrovascular resistance through a greater mean flow velocity increase per blood pressure increase in the afternoon^147^ although some studies have not demonstrated this.^145^ Furthermore, absent or reversed dipping of blood pressure is associated with impaired CVR (Figure 2).^155^

Cerebral autoregulation shares similar effector mechanisms to CVR, being dependent on vasomotor tone controlled by VSMCs. In 20 healthy volunteers, autoregulation was reduced in the morning between 6 and 12 noon, measured as the autoregulatory index from spontaneous fluctuations in blood pressure and middle cerebral artery blood flow velocity on transcranial ultrasound.^153^ There was a concurrent reduction in CVR in cerebral vessels and in flow-mediated dilatation peripherally. However, a smaller study in 16 patients did not find any diurnal variation in autoregulation but measured autoregulation as phase shift and gain from a transfer function analysis during rhythmic squat-stand maneuvers,^156^ a test designed to have improved reproducibility.^67,157^ In contrast, in an older group of patients in the 48 hours after recovery from stroke, there was an improved phase relationship at <0.05 Hz between blood pressure and CBF using transfer function analysis at rest. This was present for both the stroke and nonstroke sides of the brain and was interpreted by the authors as better autoregulation in the afternoon,^158^ but there was no significant diurnal variation in any autoregulation index in the frequency range indicative of autoregulation,^45^ but only in the frequency range more dependent on changes in carbon dioxide, indicating a difference in vasoreactivity rather than autoregulation.

Overall, empirical evidence for diurnal variation in cerebral autoregulation is limited and remains inconsistent. The autonomic component of autoregulation is associated with interindividual variation in measures of autonomic function elsewhere in the body.^45^ These do show a significant influence on circadian rhythms^159^ under the control of the SCN. Therefore, it may be hypothesized that cerebral autoregulation would also be reduced in the morning, consistent with the limited studies of systemic autonomic function such as heart rate variability.^153^

Neurovascular coupling orchestrates regional control of CBF. There are endogenous circadian rhythms in the key cell types of the neurovascular unit (VSMCs,^106,160^ endothelial cells,^161,162^ pericytes,^163^ and astrocytes), circadian variation in endothelial functions including blood-brain barrier permeability^161^ and glymphatic function,^113^ and evidence for intercellular circadian synchronicity of the components of the neurovascular unit.^163^ However, there is limited direct evidence for a circadian variation in neurovascular coupling in humans although central cellular elements in neurovascular coupling demonstrate circadian periodicity.^164^ This is a potential issue for functional magnetic resonance imaging studies that use blood flow as a proxy for neuronal function and depend on stable neurovascular coupling. However, although there is also diurnal variation in spontaneous neuronal, vascular networks in the brain^165,166^ circadian influences on neurovascular coupling have previously been felt to have minimal effects on functional magnetic resonance imaging.^167^

Overall, regulatory mechanisms for control of CBF demonstrate diurnal variation. There is reduced absolute blood flow during the night and morning, with increased morning vasomotor tone and reduced endothelial responsiveness. However, there remain limited data particularly, about neurovascular coupling and cerebral autoregulation. This is true in waking hours, where the relevance of autoregulation to the increased morning risk of cerebral ischemic and hemorrhage may be critical to understand the underlying pathophysiology and to identify treatments. However, there is virtually no data on these cerebrovascular functions during the night as opposed to the day.

### Clinical Implications of Integrated Circadian (and Diurnal) Cardiovascular Physiology

The diurnal variation in cerebrovascular events^1^ and stroke outcomes^4^ occurs in the context of prominent circadian and diurnal rhythms in nearly all aspects of systemic and cerebral cardiovascular hemodynamics. Diurnal variation in other mechanisms involved in stroke pathogenesis also contributes to this increased cerebrovascular risk,^5^ such as diurnal variation in thrombotic factors or inflammation.^168^ However, the powerful changes in hemodynamic function that correlate so closely with variation in cerebrovascular risk ensure that this is a key target to reduce a modifiable proportion of strokes.

Systemic blood pressure provides a reference to understand the interaction between circadian and diurnal variation in these mechanisms. The nadir in blood pressure at night results principally from a fall in cardiac output due to bradycardia, under the influence of endogenous circadian rhythms and descending vagal activity. This nocturnal nadir in blood pressure is largely independent of vasomotor tone but may contribute to the decline in CBF during the inactive period. This hypotensive nadir, with bradycardia and increased vagal activity, may theoretically drive an increased predisposition to stroke associated with hypotension and is a potential explanation for the apparent increased risk of lacunar stroke in patients with wake-up stroke.^15^ This may be particularly relevant in extreme dippers with a nocturnal blood pressure drop >20% who have increasingly stiff vessels^3^ and hypoperfusion in the white matter of the brain with existent small vessel disease.^169^ It is also potentially a driver of the larger core:penumbra ratio in large vessel occlusion at night.^22,23^ Nocturnal bradycardia may also increase the probability of AF, resulting in a greater proportion of embolic strokes due to cardioembolic disease than large artery disease (despite a lower overall incidence than in the morning), which may further contribute to the size of vessel occlusion and diurnal variation in core:penumbra ratios. This is likely to be exacerbated by the lower CBF at night, limiting the brain’s reserve to tolerate ischemia, but there is inadequate data about endothelial responsiveness overnight.

Before waking, the SCN-dependent release of hormones such as growth hormone and prolactin and the arousal and postural shift–driven increase in sympathomimetic hormones, such as adrenaline and noradrenaline, increase the sympathetic drive to blood vessels. This results in a sharp increase in vasomotor tone, coincident with an increase in heart rate and cardiac output, resulting in a surge in blood pressure and elevated blood pressure through the morning until lunchtime. Despite this elevation in blood pressure, CBF in humans appears not to increase until lunchtime,^170^ and this likely reflects concurrent increases in cerebrovascular tone that increases cerebrovascular resistance and reduces CBF. It is unclear whether this is part of the systemic drive to increase blood pressure to meet the demands of daytime mobility or a myogenic vasoconstrictive response secondary to the systemic rise in blood pressure.

Following the morning surge in blood pressure, there is usually a postprandial lunchtime dip in blood pressure, at a time when CBF increases, endothelial function improves, and there may be an improvement in autoregulatory capacity,^92^ after which there is on average a rise in blood pressure in the evening, which may explain the evening peak in stroke risk in some studies, before the nocturnal decline in heart rate, cardiac output, and blood pressure. However, direct measures of CBF and cerebrovascular function at this time of day are limited.

This model of the relationship between hemodynamic changes and diurnal variation in risk and recovery supposes an increased risk of hypoperfusion at night and an increased risk of hyperperfusion in the morning, explaining the morning excess risk of embolic and hemorrhagic stroke. Such a relationship between blood pressure and cerebral perfusion at specific times of day raises the possibility of chronotherapy. This provides a hypothesis that can be tested to understand the mechanism of these strokes and their outcome, subdivided by etiology. However, most studies have had inadequate phenotyping at the individual patient level of both the etiological subtype of stroke and the blood pressure phenotype (nondipping versus reverse dipping versus extreme dipping), including chronotherapy trials that did not account for relationships between individual blood pressure behavior and chronotherapy. Furthermore, some antihypertensive treatments may be better than others at normalizing maladaptive blood pressure phenotypes such as nondipping.^171,172^ Finally, in the acute setting of large vessel occlusion at times of increased risk of a poor outcome, maintenance of cerebral perfusion by limiting nocturnal blood pressure decline could be beneficial although the magnitude of any effect is likely to be far less than the benefit of reperfusion.

Diurnal variation in heart rate presents another possible chronotherapeutic target. If nocturnal bradycardia is a driver of nocturnal hypoperfusion in at-risk individuals, then prevention of excessive sinus bradycardia could be beneficial either through cessation of rate-limiting drugs such as β-blockers or changing settings in patients with a rate-responsive pacemaker. This may have the added benefit of preventing precipitation of AF at night. Although historically preventing episodes of AF was thought to have little impact on embolic risk, the recent Early Treatment of Atrial Fibrillation for Stroke Prevention Trial AF Network 4 trial suggests that a rhythm control strategy may reduce the risk of cardiovascular events.^173^

No studies have targeted diurnal variation in cerebrovascular function to improve resilience to ischemia, beyond trials of time of day of antihypertensive administration.^101^ Drugs that improve endothelial function and reduce vasomotor tone may be of particular benefit, particularly in people with an exaggerated morning surge in blood pressure, adverse nocturnal dipping behavior, or vulnerable aging cerebral circulations. Drugs targeting CVR and endothelial function may be particularly beneficial in this latter group with cerebral small vessel disease. The LACI-2 (Lacunar Intervention Trial 2)^174^ demonstrated reduced progression of cognitive dysfunction in patients with previous lacunar stroke with the vasodilating, NO donor isosorbide mononitrate, and possible improvement in functional impairment with the PDE (phosphodiesterase) 3 inhibitor cilostazol. Both drugs improve CVR,^75^ with reduced recurrent stroke rates with the addition of cilostazol to other antiplatelets,^175^ or in comparison with other antiplatelet drugs.^176,177^ Interestingly, cilostazol also increases the heart rate and reduces cerebral pulsatility, indicative of improved systemic hemodynamic function,^178^ which could, therefore, ameliorate harms associated with nocturnal bradycardia. Further trials are currently testing drugs that target the endothelium, and their interaction with diurnal variation in cerebrovascular function may improve their utility^72,179,180^ and improve outcomes for large vessel occlusions at adverse times of the day.^181^

In addition to targeting normal circadian variation in hemodynamic function and cerebral perfusion, reducing cardiovascular events is feasible through targeting abnormal circadian rhythms, whether by normalizing dysfunctional circadian rhythms such as nondipping/reverse dipping of blood pressure or minimizing the dyssynchrony between exogenous diurnal rhythms and intrinsic circadian rhythms that occur in jet lag^6^ or shift work.^31^ Although chronotherapy targeting blood pressure nonspecifically has not been shown to be beneficial, prevention of reverse dipping of blood pressure overnight and inducement of normal dipping may be beneficial. This may be achievable through treatment of obstructive sleep apnea,^182^ weight loss, or salt-depleting antihypertensives, or potentially through circadian neurohormonal interventions such as melatonin.^170^

### Recommendations for Future Research

Physiologically, the most consistent finding from this review is the relative lack of research in humans on circadian (rather than diurnal) variation in cerebrovascular function and particularly the lack of research on cerebrovascular function overnight. This research should appropriately separate circadian from diurnal influences because countermeasures for the 2 influences differ. First, there is still limited data to confirm that CVR, and associated endothelial function, has a circadian oscillation, and there are few studies specific to patients at risk of different types of stroke or with different blood pressure phenotypes. There are also no studies that have measured CVR overnight. However, this methodological challenge may be met by modern methods of continuous blood pressure and CBF measurement that could allow measurement of spontaneous fluctuations in blood pressure, CBF, and carbon dioxide that reflect endothelial function and reactivity, even during sleep.^43,183^ Opportunistic assessment of the cross-sectional diurnal variation in existing studies is feasible but will be limited to daytime changes, and the majority of studies that have measured CVR are limited in size. Finally, new studies that measure both systemic reactivity and CVR at different times of day are needed to assess whether they share underlying circadian mechanisms.

Circadian variation in cerebral autoregulation is even less well understood but may be more amenable to investigation. Although the response to a blood pressure intervention remains the optimal method of assessment of autoregulation, modern methods also use the relationship between spontaneous changes in blood pressure and CBF at rest, and so autoregulation could be more easily assessed by continuous monitoring throughout the 24-hour period. This approach has been applied in neurointensive care units in patients with head injury,^184,185^ but this population does not have normal circadian and diurnal rhythms. Furthermore, the balance of autonomic and autoregulatory mechanisms at specific frequencies measured during continuous blood pressure and CBF monitoring can provide additional information as to carbon dioxide sensitivity and the potential sympathetic versus parasympathetic balance controlling autoregulation, as well as providing specific targets for interventions to dissect underlying mechanisms.^43,67^

Systemically, there are still outstanding questions about the role of heart rate versus vasomotor tone in inducing the day-night difference in blood pressure; the balance of endogenous cellular circadian rhythms compared with external modulation; and how these mechanisms are disordered in circadian disorders and asynchrony between circadian rhythms and the environment. These questions are amenable to systematic testing in animal models, where selective neurohumoral blockade can be applied in animals exposed to continuous darkness (a free-running clock), in natural lighting conditions, or in imposed alterations in the external light signal (eg, in models of jet lag). However, these models need validating in humans, whether by selective blockade by adrenergic antagonists or opportunistic studies in patients given drugs for different reasons. The effect of heart rate can also be tested in patients with pacemakers who are routinely paced with a stable base rate without any circadian variation.

## Conclusions

Circadian and diurnal variation in CBF and its control is key to understanding diurnal variation in cerebrovascular risk, with an increased risk in the morning for most stroke subtypes but a possible further increase in lacunar infarction overnight, as well as worse physiological and clinical outcomes for nocturnal onset events. Understanding circadian and diurnal variation in CBF, however, requires an understanding not only of blood pressure but the integration of all mechanisms that determine cerebral perfusion from the heart to systemic vasomotor tone to cerebrovascular endothelial compensatory mechanisms. Extensive evidence demonstrates circadian and diurnal variation in systemic functions and blood pressure, in particular, resulting in associations between normal circadian variation, abnormal circadian function, and increased cerebrovascular risk. However, there are limited data for circadian variation in CBF and its dynamic control, which restricts understanding of the mechanisms underlying circadian variation in risk.

## ARTICLE INFORMATION

### Sources of Funding

This work was supported by the Leducq Foundation for Cardiovascular and Neurovascular Research.

### Disclosures

A.J.S. Webb received funding from the Wellcome Trust, Medical Research Council, Alzheimer’s Society, and the British Heart Foundation and received consulting fees from Woolsey Pharmaceuticals. E.B. Klerman reports consulting for the American Academy of Sleep Medicine Foundation, Circadian Therapeutics, the National Sleep Foundation, the Sleep Research Society Foundation, and the Yale University Press; travel support from the European Biological Rhythms Society, École polytechnique fédérale de Lausanne, Pavilion, the Sleep Research Society, and the World Sleep Society; and the Scientific Advisory Board (unpaid) for Chronsulting. E.B. Klerman’s partner is the founder, the director, and the chief scientific officer of Chronsulting. E.T. Mandeville reports no conflicts.
